# Gut microbiota and viral respiratory infections: microbial alterations, immune modulation, and impact on disease severity: a narrative review

**DOI:** 10.3389/fmicb.2025.1605143

**Published:** 2025-07-18

**Authors:** Gaelle El-Khoury, Crystel Hajjar, Regina Geitani, Dolla Karam Sarkis, Marie-José Butel, Frédéric Barbut, Marianne Abifadel, Nathalie Kapel

**Affiliations:** ^1^Laboratory of Microbiology, Faculty of Pharmacy, Saint Joseph University of Beirut, Beirut, Lebanon; ^2^INSERM S-1139, Université Paris Cité, Paris, France; ^3^FHU PREMA Fighting Prematurity, Paris, France; ^4^FHU PaCeMm, Paris Center for Microbiome Medicine, Paris, France; ^5^Laboratory of Biochemistry and Molecular Therapeutics (LBTM), Faculty of Pharmacy, Pôle Technologie-Santé, Saint Joseph University of Beirut, Beirut, Lebanon; ^6^INSERM, Foundation for Innovation in Cardiometabolism and Nutrition (ICAN), UMRS 1166, Sorbonne Université, Paris, France; ^7^Service de Coprologie Fonctionnelle, APHP, Hôpital Universitaire Pitié Salpêtrière, Paris, France

**Keywords:** SARS-CoV-2, influenza, RSV, respiratory viruses, gut microbiota, gut-lung axis

## Abstract

Respiratory viral infections are a major public health concern, accounting for millions of infections annually and contributing significantly to global morbidity and mortality. Influenza and respiratory syncytial virus (RSV) have long been recognized as critical pathogens, while the recent emergence of severe acute respiratory syndrome coronavirus 2 (SARS-CoV-2) has led to the COVID-19 pandemic. These viruses typically affect both the upper and lower respiratory tracts and can cause a broad spectrum of clinical manifestations, ranging from mild symptoms to severe respiratory failure and multi-organ dysfunction. Gastrointestinal symptoms are also frequently reported, suggesting a potential link between respiratory viruses and gut microbiota alterations. This connection highlights the role of the gut microbiota in disease pathophysiology. This narrative review summarizes current evidence on gut microbiota changes associated with SARS-CoV-2, influenza, and RSV infections. It further explores the microbiota’s role in immune regulation and host homeostasis, and discusses the potential of microbiota-targeted strategies in the prevention and management of acute respiratory syndromes.

## Background

Respiratory viral infections have historically been a leading cause of global morbidity and mortality, with outbreaks, epidemics, and pandemics affecting human populations throughout history. These infections account for approximately one-fifth of childhood deaths worldwide. Common respiratory viruses include adenovirus, enterovirus, metapneumovirus, rhinovirus, parainfluenza virus, influenza virus, coronavirus and respiratory syncytial virus (RSV) (Hodinka, 2016). Among these, coronaviruses, influenza viruses, and RSV are the most extensively studied. Coronaviruses are closely monitored due to their pandemic potential, influenza viruses are linked to pneumonia-related deaths, and RSV is the leading cause of hospitalizations due to respiratory infections in infants. Coronaviruses were first identified in chicken embryos in 1937. Interest in this family of viruses surged in the early 21st-century with the outbreaks of severe acute respiratory syndrome (SARS-CoV) in China and Middle East respiratory syndrome (MERS-CoV) in Saudi Arabia (Ludwig and Zarbock, 2020). More recently, the emergence of severe acute respiratory syndrome 2 (SARS-CoV-2) triggered the ongoing global pandemic Coronavirus disease 2019 (COVID-19), resulting in millions of infections and high mortality, especially among the elderly and individuals with comorbidities (Wiersinga et al., 2020). In addition to its health impact, the pandemic has had a profound impact on mental health and global economic stability. Meanwhile, seasonal influenza epidemics arise annually, causing between 290,000 and 645,000 death globally each year (Iuliano et al., 2018). RSV remains the leading cause of lower respiratory tract infections, such as bronchiolitis and pneumonia, especially in children under 1 year old with approximately 33 million cases and 3.4 million hospitalizations annually (Nair et al., 2010). Regardless of the specific viral pathogen, infected individuals typically present with mild to moderate symptoms, including fever, headache, fatigue, sore throat, cough, nasal congestion, and myalgia. Severe cases may progress to hypoxemia, pneumonia, hyperinflammation, sepsis, and multi-organ failure (Wiersinga et al., 2020). Notably, gastrointestinal (GI) symptoms, such as abdominal pain, nausea, diarrhea, and vomiting, are frequently observed, with prevalence depending on the virus and study population. For instance, diarrhea occurs in 4–25% of SARS-CoV-2 cases (Cheung et al., 2020; Guan et al., 2020), and in around 15% of influenza and RSV infections (Newman et al., 2023). These observations highlight a potential link between respiratory infections and alterations of the gut microbiota (GM). Furthermore, patients with inflammatory bowel disease frequently exhibit impaired lung function despite the absence of overt respiratory illness (Enaud et al., 2020; Zhou et al., 2021). Emerging evidence suggests that GM imbalance may increase susceptibility to respiratory viral infections (Enaud et al., 2020), while respiratory inflammation may in turn disrupt gut microbial composition (Xiao et al., 2023). This bidirectional communication between the respiratory tract and the gut is known as the “gut-lung axis.” In this review, we focus on the progression and severity of viral respiratory illnesses and their correlation with GM composition, with an emphasis on the immune-regulatory functions of the GM.

## The gut-lung axis

**Figure 1:**
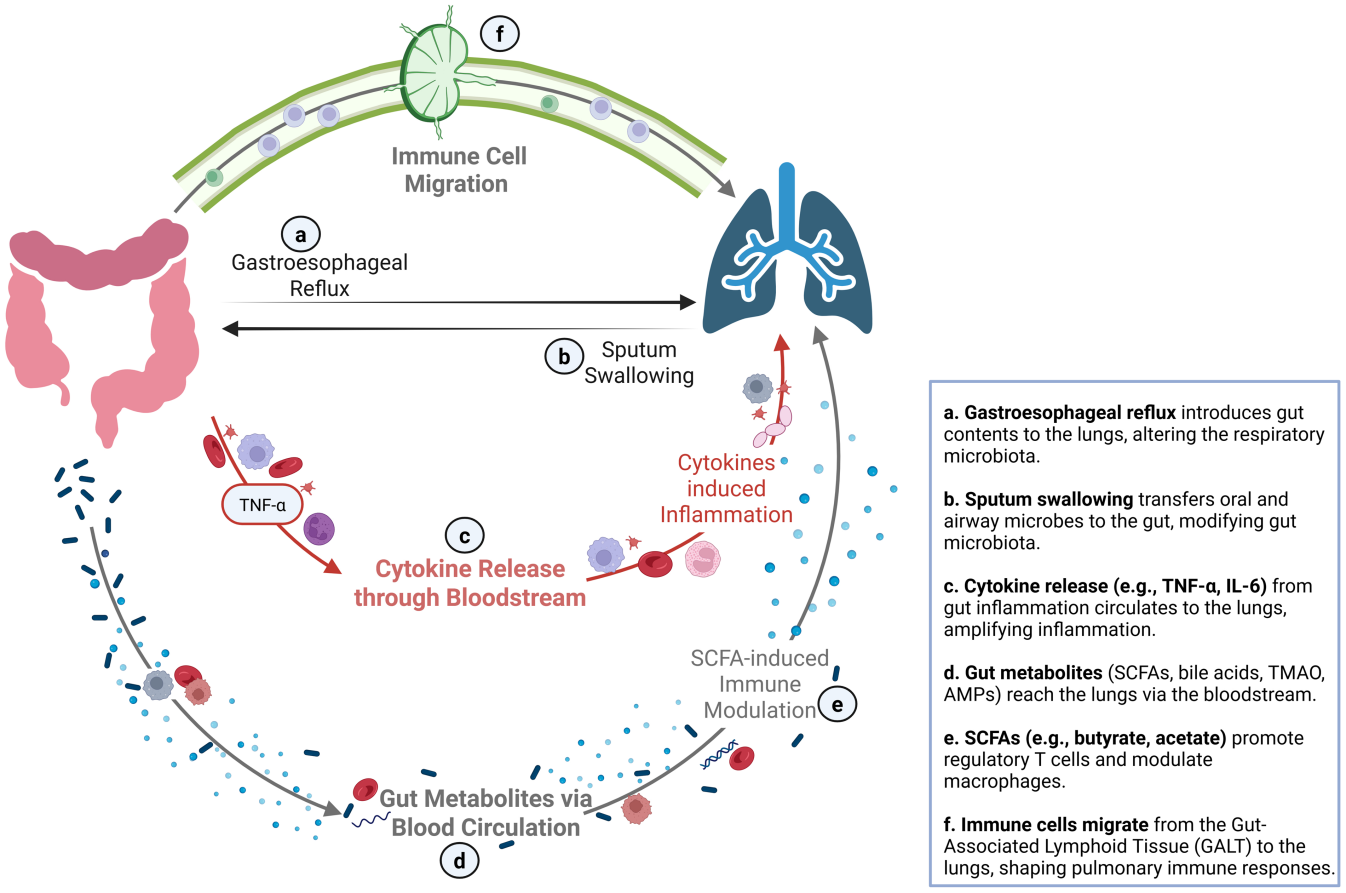
The gut–lung axis: bidirectional communication through microbial, immune, and metabolic pathways. This figure highlights the key mechanisms connecting the gastrointestinal tract and lungs. The gut–lung axis operates via direct routes such as microbial exchange and reflux, and indirect routes involving systemic circulation of cytokines, metabolites, and immune cells. Inflammatory gut signals can exacerbate pulmonary inflammation, while gut-derived metabolites and immune-regulatory circuits support respiratory homeostasis. This inter-organ crosstalk plays a critical role in both health and disease, with implications for infections, inflammatory airway conditions, and immune modulation. SCFA, short-chain fatty acid; AMP, antimicrobial peptide; TMAO, trimethylamine N-oxide; TNF-*α*, tumor necrosis factor alpha; IL, interleukin; Treg, regulatory T cell; GALT, gut-associated lymphoid tissue.

The gut–lung axis refers to the bidirectional interaction between the GI and respiratory systems, primarily mediated by the GM. The key mechanisms connecting the GI tract and lungs are presented in Figure 1. The GM is a highly complex and dynamic ecosystem comprising bacteria, archaea, eukaryotes, and viruses that have co-evolved with the host to establish mutually beneficial relationships. It comprises approximately 10^13^ microorganisms, a number comparable to that of human cells. Although it encompasses various microbial kingdoms, the community is dominated by bacteria, and the term “gut microbiota” usually refers to the bacterial component. The bacterial GM is predominantly composed of four phyla: *Bacillota* (*Firmicutes*), *Bacteroidota* (*Bacteroidetes*), *Pseudomonadota* (*Proteobacteria*), and *Actinomycetota* (*Actinobacteria*). Its diversity is shaped by intrinsic and extrinsic factors including age, environment, diet, lifestyle, and antibiotic use (Jandhyala et al., 2015). The GM plays an essential role in mucosal immune development and regulation, supporting both innate and adaptive immune responses. Approximately 70% of immune cells are found in the mucosa-associated lymphoid tissue (MALT), which is a critical network that connects mucosal immunity throughout the gut, lungs, and other surfaces (Enaud et al., 2020). The GM directly modulates immune responses, namely antiviral responses, by influencing neutrophil activity, Toll-like receptor (TLR) signaling, and the production of proinflammatory or regulatory cytokines. It also supports the differentiation of CD4 + T and CD8 + T cells into T helper (Th) 1, Th2, Th17, and regulatory T (Treg), as well as B cells via microbial-associated molecular patterns (MAMPs) such as peptidoglycan, lipopolysaccharides, and flagellin (Enaud et al., 2020; Baradaran et al., 2021). Moreover, GM-derived fermentation products, particularly short-chain fatty acids (SCFAs) such as butyrate, acetate, and propionate, which together represent 90–95% of total SCFAs, play a key role in shaping immune responses by binding to G-protein-coupled receptors on dendritic cells and promoting the release of cytokines such as TGF-*β* and retinoic acid. This process induces IgA class switching and the differentiation of B cells into IgA-secreting plasma cells. SCFAs also downregulate inflammatory cytokines such as TNF-*α* and strengthen intestinal barrier integrity (Baradaran et al., 2021; Li et al., 2022; Rastogi et al., 2022; Alswat, 2024). Finally, the GM plays a key role in maintaining intestinal and systemic immune homeostasis by limiting microbial translocation and producing antimicrobial peptides (Alswat, 2024).

Once believed to be sterile, it is now recognized that the lungs host a microbiota, albeit with a much lower density than the gut. This community is thought to be transient and continuously replenished via inhalation, with subsequent clearance through mucociliary mechanisms. It predominantly comprises the same phyla as the GM, mainly *Bacteroidota* and *Bacillota*, with a density estimated at 10–100 bacteria per 1,000 human cells (Enaud et al., 2020; Rastogi et al., 2022; Marrella et al., 2024). Like the GM, the lung microbiota evolves with age, gaining diversity and functional capacity, and interacts closely with the host immune system. It thus contributes to immune surveillance, epithelial barrier maintenance, and protection against respiratory pathogens through crosstalk with resident immune cells (Marrella et al., 2024).

The gut and the lungs are connected by both direct and indirect pathways. Direct routes include the swallowing of infected sputum or the aspiration of gastroesophageal contents. Indirect communication is mediated by immune signaling and systemic dissemination of microbial metabolites, cytokines, and bacterial fragments (Enaud et al., 2020; Zhou et al., 2021; Baradaran et al., 2021). Components of the GM, such as SCFAs, have a central role. They can enter the bloodstream and modulate distant immune compartments, including the lungs. SCFAs exert anti-inflammatory effects by modulating immune cell migration and suppressing NF-κB activation pathways. SCFAs also promote hematopoiesis in the bone marrow and influence the immune microenvironment in the lungs by enhancing the generation of macrophage and dendritic cell precursors, followed by the seeding of the lungs with dendritic cells that exhibit high phagocytic activities (Trompette et al., 2014). SCFAs also dampen lung inflammation by reducing Th2 cell responses and stimulating Treg cell activity (Trompette et al., 2014). Ultimately, the GM plays a critical role in balancing immune tolerance toward commensal microorganisms while maintaining an effective immune response against invading pathogens. Studies have shown that antiviral responses of CD4 + and CD8 + T cells, as well as B cells are modulated by the GM. An imbalanced GM can disrupt cytokines release and impair dendritic cells migration in influenza-infected mice (Ahmadi Badi et al., 2021). Conversely, respiratory viral infections such as influenza have been shown to alter the GM by reducing microbial diversity and affecting SCFAs production, which may contribute to secondary pathogen overgrowth and inflammation (Enaud et al., 2020; Kalam and Balasubramaniam, 2024). Immune cells, cytokines, and microbial metabolites can thus travel via the blood and lymphatic systems from gut-associated lymphoid tissue (GALT) to bronchial-associated lymphoid tissue (BALT), reinforcing host defenses against respiratory infections (Enaud et al., 2020; Ahmadi Badi et al., 2021). Using a mouse model, Wang et al. demonstrated that lung-derived T cells infected by a respiratory virus can migrate to the small intestine and alter the GM through interferon-*γ* signaling. These effects may predispose to secondary enteric infections (Wang et al., 2014). Thus, gut dysbiosis may worsen respiratory illness severity, while lung infections can impair gut health, highlighting a bidirectional and dynamic gut–lung axis. Although the underlying mechanisms are not yet fully elucidated, this axis likely plays a central role in shaping disease presentation and outcome. This has prompted growing interest in understanding GM alterations during respiratory viral infections and their potential as therapeutic targets (Gu et al., 2020; Zuo et al., 2020; Tao et al., 2020; Wu et al., 2021; Khan et al., 2021; Li et al., 2021; Yeoh et al., 2021; Mazzarelli et al., 2021; Gaibani et al., 2021; Newsome et al., 2021; Cao et al., 2021; Hazan et al., 2022; Xu et al., 2022; Nagata et al., 2023; de Nies et al., 2023; Xu et al., 2021; Romani et al., 2022; Nashed et al., 2022; Suskun et al., 2022; Gutiérrez-Díaz et al., 2023; Qin et al., 2015; Gierse et al., 2021; Chen et al., 2023; Harding et al., 2020; Groves et al., 2018; Groves et al., 2020).

## Insights into gut microbiota alterations during viral respiratory infections: COVID-19, influenza, and RSV

### COVID-19

#### Studies in adults

Several studies have reported alterations in GM composition among COVID-19 patients compared to healthy adults. The gut microbial profile in COVID-19 patients is generally characterized by reduced microbial diversity, decreased bacterial richness, enrichment of opportunistic pathogens, and depletion of beneficial bacteria (Gu et al., 2020). In particular, butyrate-producing bacteria such as *Roseburia*, *Coprococcus* (Zuo et al., 2020; Tao et al., 2020), *Lachnospira* (Zuo et al., 2020; Wu et al., 2021; Khan et al., 2021; Li et al., 2021), *Ruminococcus* (Khan et al., 2021), and *Faecalibacterium* (Tao et al., 2020; Yeoh et al., 2021), seem to be consistently diminished in infected patients. Additionally, the absence of *Butyricicoccus pullicaecorum*, *Clostridium ruminatium*, *Lachnospira pectinoschiza*, and *Pseudobutyrivibrio xylanivorans* has been associated with infected individuals, distinguishing them from healthy controls (Wu et al., 2021; Khan et al., 2021). In contrast, the presence of some specific bacterial species has been proposed as potential biomarkers for COVID-19. For instance, *Streptococcus thermophilus*, *Bacteroides oleiciplenus*, *Fusobacterium ulcerans*, and *Prevotella bivia* have been detected exclusively in COVID-19 patients, suggesting their potential as indicators of SARS-CoV-2 infection. Interestingly, the GM profiles of SARS-CoV-2 patients also differ from those observed in influenza patients, suggesting the presence of virus-specific dysbiotic patterns (Gu et al., 2020; Mazzarelli et al., 2021).

#### Studies in children

**Figure 2:**
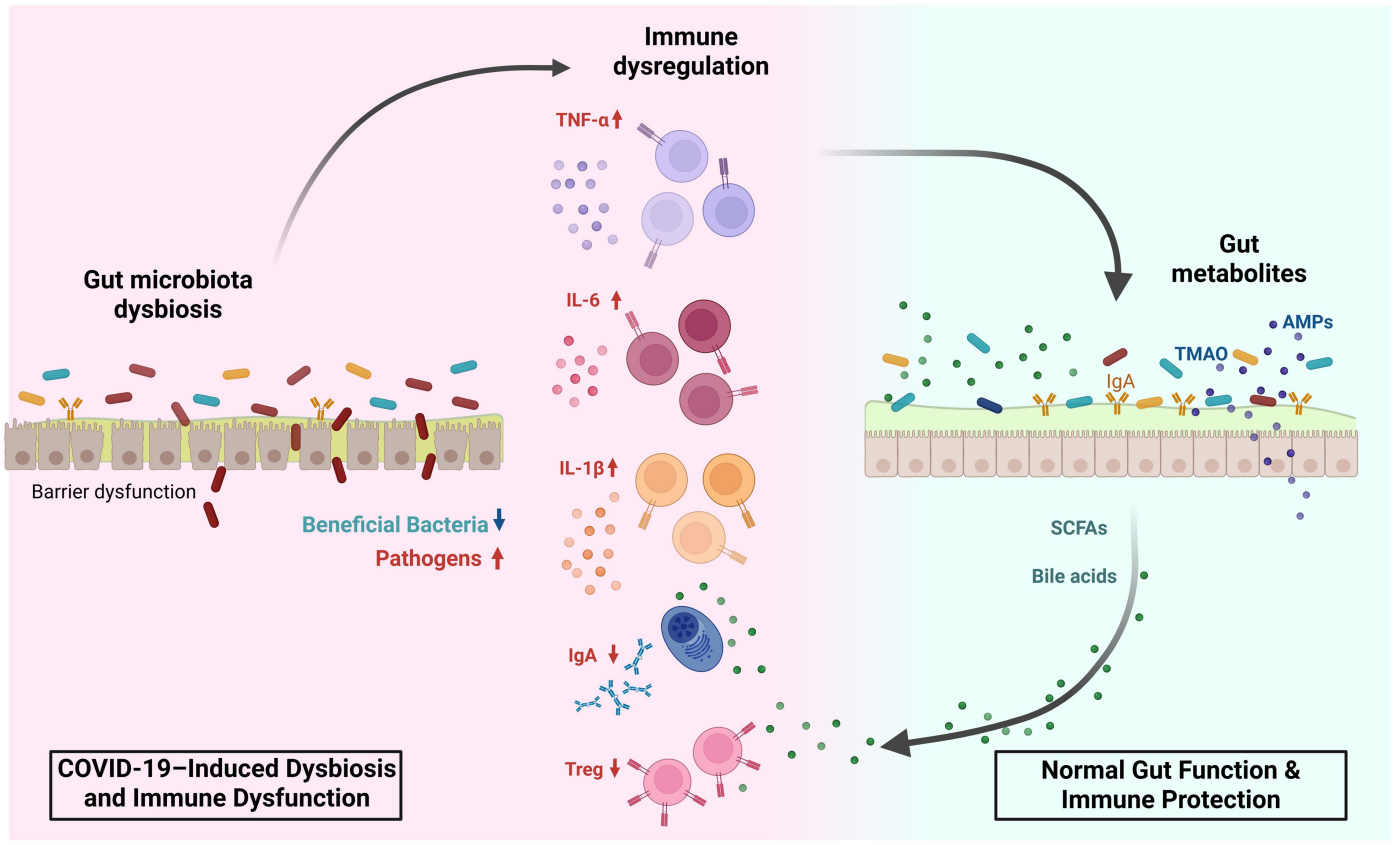
Gut microbiota dysbiosis and immune modulation in SARS-CoV-2 infection. This figure illustrates how SARS-CoV-2–associated gut dysbiosis (left, red background) contrasts with normal gut function (right, blue background) in shaping host immunity. On the left, COVID-19-induced dysbiosis involves a reduction in beneficial bacteria (e.g., *Faecalibacterium prausnitzii*, *Bifidobacterium*, and *Roseburia*) and expansion of pathogenic taxa (e.g., *Streptococcus*, *Prevotella*, and *Parabacteroides*). This imbalance leads to mucosal barrier dysfunction, overproduction of inflammatory cytokines (TNF-α, IL-6, IL-1*β*), decreased IgA secretion, and reduced Treg cell differentiation, driving immune dysregulation. On the right, a healthy gut microbiota supports immune homeostasis through production of short-chain fatty acids (SCFAs), bile acids, trimethylamine N-oxide (TMAO), and antimicrobial peptides (AMPs). These metabolites maintain epithelial integrity, promote IgA and Treg responses, and regulate inflammation. This balance between dysbiosis-driven immune activation and metabolite-mediated protection plays a key role in modulating COVID-19 severity and systemic inflammation. SCFA, short-chain fatty acid; TMAO, trimethylamine N-oxide; AMP, antimicrobial peptide; TNF-α, tumor necrosis factor alpha; IL, interleukin; IgA, immunoglobulin A; Treg, regulatory T cell.

Studies in pediatric populations remain limited, with results varying due to differences in participants’ ages. Nonetheless, the GM of infected children generally exhibits a dysbiotic state, characterized as in adults, by a depletion of butyrate-producing and inflammation-preventing bacteria such as *Blautia*, *Coprococcus*, *Ruminococcus* (Romani et al., 2022), *Bifidibacterium bifidum,* and *Akkermansia muciniphila* (Nashed et al., 2022; Zhang et al., 2023), along with an enrichment in pathogenic bacteria like *Neisseria* (Romani et al., 2022) and *Pseudomonas* (Hazan et al., 2022). Overall, both adults and children with COVID-19 shows an increased pathogenic-to-commensal bacteria ratio and a heightened inflammatory tendency due to a reduction in SCFA-producing bacteria with anti-inflammatory properties (Figure 2). A comprehensive summary of GM dysbiosis in these patients is provided in Tables 1, 2. However, given the heterogeneity of study cohorts, which differ in terms of age, ethnicity, comorbidities, and are often limited in sample size, it remains challenging to draw definitive conclusions or to define a specific microbial profile for patients with COVID-19.

**Table 1:** Summary of studies on the alterations of gut microbiota composition in adults patients following SARS-CoV-2 infection.

Study, country, year of publication	Participants	Summary of key findings	Limitations
Gu et al. (2020), China	30 COVID-19 patients 24 influenza patients 30 healthy controls	Selection of five biomarkers to distinguish COVID-19 patients from controls: *Fusicatenibacter*, *Romboutsia*, *Intestinibacter*, *Actinomyces*, *Erysipelatoclostridium*	Single-center study Small sample size Non evaluation of patients at different disease stages Presence of different parameters that can alter the GM composition
Zuo et al. (2020), China	15 COVID-19 patients (44–67.5 years) 6 community-acquired pneumonia control group (44–65 years) 15 healthy controls (45–48 years)	Depletion of symbionts and enrichment of opportunistic pathogens in COVID-19 patients Association between GM composition and disease severity Negative correlation between some *Bacteroides* species and SARS-CoV-2 fecal shedding	Small sample size Limited generalizability due to inclusion of only hospitalized moderate/severe COVID-19 cases
Tao et al. (2020), China	62 COVID-19 patients 33 influenza patients 40 healthy controls	Association between increased *Streptococcus* abundance and risk of infection by opportunistic pathogenic bacteria in COVID-19 patients Negative correlation between *Bilophila, Citrobacter* and disease severity Unique microbiota pattern: *Helicobacter* in COVID-19 patients compared with healthy controls	Small sample size Potential effect of medical treatment on GM
Wu et al. (2021), China	13 COVID-19 patients 15 pneumonia controls 15 healthy controls	Associated shift between gut and upper airway microbiota Inter-personal and inter-timepoint variations of GM	Small sample size Incomplete data collection
Khan et al. (2021), India	30 COVID-19 patients: *Asymptomatic:* 58 ± 18 years *Mild*: 49 ± 13 years *Severe*: 46 ± 9 years 10 healthy controls	Domination of *Lachnospiraceae* and *Ruminococcceae* in healthy guts Increase in *Bacteroidota* with disease progression from asymptomatic to severe stages accompanied by a reduction in the relative abundance of *Bacillota* Fivefold increase of *Bifidobacterium* count in the severely infected group	Small sample size Lack of medication records before hospital admission Limited generalizability due to regional dietary patterns
Li et al. (2021), China	*Discovery cohort*: 37 COVID-19 patients (44 ± 15 years) 10 healthy controls (37 ± 9 years) *Validation cohort:* 10 COVID-19 patients (56 ± 14 years) 9 healthy controls (47 ± 15 years)	Identification of 4 unique microorganisms in COVID-19 patients: *Streptococcus thermophilus*, *Bacteroides oleiciplenus*, *Fusobacterium ulcerans*, and *Prevotella bivia* Negative correlation between *Roseburia inulinivorans*, *Bacteroides faecis*, *Bifidobacterium bifidum*, *Parabacteroides goldsteinii*, *Lachnospiraceae* bacterium 9143BFAA, and *Megasphaera* sp. and COVID-19 severity. Positive correlation between *Paraprevotella* sp., *Streptococcus thermophilus*, *Clostridium ramosum*, and *Bifidobacterium animalis* and COVID-19 severity	Small sample size Uneven distribution of patients according to disease severity One time point collection of samples
Yeoh et al. (2021), China	100 COVID-19 patients, including 27 recovered patients (36 ± 19 years) 78 healthy controls (46 ± 13 years)	Negative correlation between *Faecalibacterium prausnitzii* and *Bifidobacterium bifidum* and disease severity Association between GM composition and immune response Persistence of dysbiosis after SARS-CoV-2 clearance	Short patients follow up Heterogeneous patient clinical management Heterogeneity of GM across populations
Mazzarelli et al. (2021), Italy	9 COVID-19 patients in infectious disease wards (44–83 years) 6 COVID-19 patients in ICU; (64–74 years) 8 controls (51–77 years) All participants had concomitant pneumonia	Distinct GM profiles between ICU versus ward COVID-19 patients (the latter being closer to controls)	Single center study Small sample size Potential impact of ICU stays on GM composition Potential effect of antibiotic treatment on GM Use of rectal swabs instead of standard fecal samples for GM analysis
Gaibani et al. (2021), Italy	69 COVID-19 hospitalized patients (59–85 years) Age- and sex- matched healthy controls	Increase in opportunistic pathogens: *Enterococcus*, *Staphylococcus*, *Serratia*, *Collinsella*, *Lactobacillus*, *Parabacteroides*, *Lactococcus*, *Phascolarctobacterium*, *Odoribacter*, *Actinomyces*, *Methanobrevibacter* and *Akkermansia* in patients Association between *Streptococcus*, *Oscillospira*, *Blautia*, *Ruminococcacea*, *Lachnospiraceae*, and *Clostridiales* taxa and non- ICU admission and non-bloodstream infection development Positive correlation between *Enterococcus* and ICU admission	Small sample size Non standardized bacterial superinfection diagnosis and therapy protocols Hospitalization in several ICUs Lack of a non-COVID-19 hospitalized control group Multiple GM-associated confounders such as ATB intake
Newsome et al. (2021), USA	50 COVID-19 patients (mean age 62 years) 9 COVID-19 recovered patients (mean age 47 years) 34 controls (mean age 55 years)	Association between SARS-CoV-2 infection and increased relative abundance of *Campylobacter* and *Klebsiella* Similar GM composition between recovered patients and non-infected patients	Small sample size Use of different media for fecal sample collection
Cao et al., (2021), China	13 COVID-19 patients (8 ATB naive patients and 5 ATB-treated patients) (15–85 years) 5 healthy controls	Enrichment of *Corynebacterium durum*, *Rothia mucilaginosa*, *Enterococcus faecium*, and *Campylobacter gracilis* in severe COVID-19 cases Enrichment of *Eubacterium rectale* in mild cases of COVID-19 Enrichment of *Corynebacterium*, *Enterococcus*, *Rothia*, *Megasphaera*, and *Campylobacter*; depletion of *Eubacterium* in severe cases Additional microbial shifts in ATB-treated COVID-19 patients: decrease in butyrate-producing bacteria (*Roseburia hominis* and *Faecalibacterium prausnitzii*)	Small sample size Confounding effect of ATBs on microbiome analysis
Hazan et al. (2022), USA	50 COVID-19 patients (50 ± 3 years) 20 PCR-negative exposed controls (44 ± 4 years)	Negative correlation between diversity and SARS-CoV-2 severity Association between increased disease severity and decreased relative abundance of *Bifidobacterium*, *Faecalibacterium*, *Faecalibacterium prausnitizii* and *Roseburium*, and increased relative abundance of *Bacteroides*	Small sample size
Xu et al. (2022), China	38 COVID-19 patients: *Severe/Critical*: *n* = 14 (49–71 years) *Mild/Moderate*: *n* = 24 (43–56 years) 31 healthy controls	Suppression of *Lachnospira eligens, Klebsiella pneumoniae,* and *Roseburia intestinalis* in severe/critical COVID-19 patients Increase in *Akkermansia muciniphila*, *Bacteroides ovatus*, and *Bacteroides cellulosilyticus* in severe/critical COVID-19 patients	Small sample size
Nagata et al. (2023), Japan	112 COVID-19 hospitalized adult patients (>20 years) 112 controls matched by baseline factors, recruited before the pandemic	No difference in α diversity; dissimilarities in β diversity (COVID-19 vs. controls; mild vs. severe COVID-19) Depletion in *Bifidobacterium*, *Dorea*, *Roseburia* and *Butyricicoccus* in COVID-19 patients, and *Methanobrevibacter smithii* in severe COVID-19 and pneumonia groups Enrichment in *Ruminococcus torques* in COVID-19 patients Association between the levels of amino acids, carbohydrates, and neurotransmitters and gut microbes Association between microbial and metabolomic alterations and pulmonary complications	Lack of a non-COVID-19 control group with severe illness to control for confounders
de Nies et al. (2023), Luxembourg	61 asymptomatic-to-moderate COVID-19 (43.85 ± 11.92 years) 57 controls (42.12 ± 3.32 years)	No difference in α and β diversity between COVID-19 patients and controls Decrease in CAG 145 (*Bacillota* phylum), *Roseburia faecis*, and *Turicibacter sanguinis* in COVID-19 Increase in *Lachnospiraceae*, *Ruminococcaceae*, *Bacteroidaceae*, and *Bifidobacteriaceae* species in COVID-19 Increase in AM10 47 (*Bacillota* phylum*), Prevotella* sp. CAG 520, *Prevotella stercorea* and *Roseburia* sp. CAG 471 in the COVID-19 group No significant changes in the functional profile of the microbiome between COVID-19 and control groups	Small sample size

ATB, antibiotic; COVID-19, coronavirus disease 2019; GM, gut microbiota; ICU, intensive care unit; SARS-CoV-2, severe acute respiratory syndrome coronavirus 2.

**Table 2:** Summary of studies on the gut microbiota dysbiosis in children patients following SARS-CoV-2 infection.

Study, country, year of publication	Participants	Summary of key findings	Limitations
Xu et al. (2021), China	9 COVID-19 patients (1–139 months) 14 controls	Persistence of pathogenic bacteria *Pseudomonas veronii* in COVID-19 children Persistence of dysbiosis after recovery	Small sample size
Romani et al. (2022), Italy	68 COVID-19 patients (1–12 years) 16 non-COVID-19 patients (2–6 years) 4 patients with multisystem inflammatory syndrome (10–13 years) 95 controls	Selection of microbial markers for COVID-19: *Staphylococcus*, *Anaerostipes*, *Faecalibacterium*, *Dorea*, *Dialister*, *Streptococcus*, *Roseburia*, *Haemophilus*, *Granulicatella*, *Gemmiger*, *Lachnospira*, *Corynebacterium*, *Prevotella*, *Bilophila*, *Phascolarctobacterium*, *Oscillospira*, and *Veillonella* Identification of *Faecalibacterium* as a marker of pediatric COVID-19	Small sample size Limited number of children with multisystem inflammatory syndrome Potential confounding effect of ATBs on GM profiling
Nashed et al. (2022), USA	13 COVID-19 patients (0–2 years) 26 controls (0–2 years)	Detection of microbiome changes in COVID-19 asymptomatic infants Decrease in anti-inflammatory taxa in COVID-19 patients	Small sample size
Suskun et al., (2022), Turkey	20 COVID-19 patients (5 – 11 years) 25 patients with a multisystem inflammatory syndrome (5–10 years) 19 controls (7–10 years)	Dominance of *Bacteroides coprophilus*, *Bifidobacterium adolescentis*, *Dorea formicigenerans*, *Ruminococcus albus*, and *Clostridium piliforme* in the COVID-19 group	Small sample size Single fecal sample at diagnosis Enrolment during global COVID-19 restrictions, potentially influencing GM composition
Gutiérrez-Díaz et al. (2023), Spain	19 COVID-19 patients (0–2 years) 18 age-matched controls	Decrease in *Actinobacteria*, *Bifidobacteriacea*, and *Bifidobacterium breve* in COVID-19 children Increase in *Enterobacteriaceae* in COVID-19 patients Non-significant microbial differences between patients with gastrointestinal symptoms and controls or patients without digestive manifestations, with a trend of increased *Bacteroidota* and decreased *Actinomycetota*	Small sample size Potential bias due to medical treatment received by patients

COVID-19, coronavirus disease 2019; GM, gut microbiota.

### Influenza and respiratory syncytial virus

#### Studies in humans

Although few studies have examined GM alterations during influenza infection in humans, available data suggest a pattern similar to that observed in patients with SARS-CoV-2, characterized by reduced microbial diversity and richness, alongside a depletion of immunomodulatory bacteria such as *Faecalibacterium, Ruminococcus*, *Bifidobacterium*, and *Roseburia*, as well as an increase in pathobionts such as *Escherichia*, *Shigella*, *Enterococcus*, and *Salmonella* (Gu et al., 2020; Chen et al., 2023; Table 3). No specific data are available for the pediatric population.

**Table 3:** Overview of research findings on gut microbiota alterations during influenza infection in humans and animals.

Study, country, year of publication	Participants	Summary of key findings	Limitations
Qin et al. (2015), China	26 influenza patients: (30–80 years) 31 age- and sex-matched healthy controls	Greater reduction in microbial diversity among ATB-treated patients Higher abundance of *Escherichia coli* in ATB-treated patients, potentially correlating with infection progression Dramatical decrease in *Faecalibacterium prausnitzii* in ATB-treated group Unstable GM in patients compared to stable GM in healthy individuals Enrichment in *Roseburia inulinivorans*, butyrate-producing bacteriumSS3/4, *Eubacterium ventriosum*, *Roseburia intestinalis*, and *Ruminococcus* in controls Enrichment in *Clostridium*, *Enterococcus*, *Enterobacter* and *Clostridium butyricum* in the H7N9 patients	Fecal samples were not collected from patients in the early stages of infection No stool samples were collected from patients after discharge to assess long-term effects
Gu et al. (2020), China	30 COVID-19 patients 24 influenza patients 30 matched healthy controls	Selection of six biomarkers to distinguish between the influenza group and healthy controls: *Fusicatenibacter*, *Romboutsia*, *Anaerostipes*, *Eubacterium hallii* group, *Ruminococcus torques* group, and *Blautia*	Single-center study Small sample size Non evaluation of patients at different disease stages Presence of different parameters that can alter the GM composition
Gierse et al. (2021), Germany	3 influenza A infected pigs: 11 weeks 3 healthy pigs	Alteration of the taxonomic composition of the GM. Reduction in *Streptococcaceae* as a possible indicator of influenza A infection in pigs	Limited biomass obtained from nasal swabs
Chen et al. (2023), China	20 female mice	Decrease in *Lactobacillus murinus* as a biomarker of influenza A infection	The use of murine models may limit the applicability of findings to human due to coprophagy in mice

ATB, antibiotic; GM, gut microbiota.

In the case of RSV, Harding et al. reported no difference in species richness or alpha diversity between patients with RSV and controls. However they reported a marked alteration in the GM composition, with an increase in the families S24_7, *Clostridiales*, *Odoribacteraceae*, *Lactobacillaceae*, and *Actinomyces* in infected children (Harding et al., 2020).

#### Animal studies

In animal models, both influenza and RSV infection have been shown to induce significant GM dysbiosis. In mice and swine, influenza infection was associated with increased GM diversity and richness (Harding et al., 2020; Groves et al., 2018). Notably, the *Streptococcaceae* family was significantly depleted in infected swine, suggesting its potential as a microbial marker of influenza infection in pigs (Gierse et al., 2021). Similarly, RSV-infected mice displayed an increased *Bacteroidota* to *Bacillota* ratio (Groves et al., 2020). This dysbiosis may be attributed to increased mucus production during infection, which serves as an energy source for certain bacteria, as well as reduced food intake due to infection-induced inappetence (Groves et al., 2018; Groves et al., 2020). Table 4 summarizes the observed GM dysbiosis during RSV infection.

**Table 4:** Summary of studies on RSV-induced gut microbiota changes in humans and animals.

Study, country, year of publication	Participants	Summary of key findings	Limitations
Harding et al. (2020), USA	58 RSV patients *Moderate*: *n* = 53 (median age: 94 days) *Severe*: *n* = 5 (median age: 60 days) 37 controls (median age: 93 days)	Association between S24_7, *Odoribacter*, and *Oribacterium* and RSV severity Association between *Clostridiales* and *Coriobacteriaceae* and moderate disease	Small sample size, particularly for severe RSV patients Socioeconomic and racial skew in patient demographics limiting generalizability
Groves et al. (2018), UK	10–12 weeks female infected and naive mice	Alteration of the GM with an enrichment in *Bacteroidota*	Unclear translational impacts of the findings
Groves et al. (2020), UK	Adult female mice	Similarity in GM changes after either RSV or influenza A infection, implying a common mechanism to both infections	Unclear translational impacts of the findings

GM, gut microbiota; RSV, respiratory syncytial virus.

## Consequences of intestinal dysbiosis

Intestinal dysbiosis can exacerbate respiratory viral infections by influencing viral entry mechanisms, modulating immune responses, and worsening disease severity.

### Facilitation of viral entry

Although the direct effects of the GM on influenza and RSV viral receptors remain poorly studied, increasing evidence points to a regulating role of the GM in modulating the expression of angiotensin-converting enzyme 2 (ACE2), the cellular entry receptor for SARS-CoV-2. ACE2 is expressed in multiple organs, including the respiratory tract, GI epithelium, kidneys, heart, liver, testes, and brain, with notably high levels in the intestinal mucosa. Beyond serving as a viral entry receptor, ACE2 plays key physiological roles, including the regulation of the renin-angiotensin system by converting angiotensin II into angiotensin (1-7), thereby promoting vasodilation and exerting anti-inflammatory effects. It also contributes to intestinal homeostasis by modulating inflammation and amino acid absorption. After SARS-CoV-2 binds to ACE2, transmembrane serine protease 2 (TMPRSS2) cleaves the receptor to facilitate viral entry, thereby impairing its physiological functions (Zuo et al., 2020; Li et al., 2020). Interestingly, the expression of ACE2 is, at least in part, modulated by the GM as some species within the *Bacteroides* genus, such as *B. dorei*, *B. thetaiotaomicron*, *B. massiliensis*, and *B. ovatus*, have been shown to downregulate ACE2 expression in murine colonocytes, potentially limiting viral entry and correlating with less severe COVID-19 outcomes (Zuo et al., 2020).

### Disease severity

Several studies suggest that the severity of viral infections, particularly those caused by SARS-CoV-2, may be more closely related to the host’s immune response than to the viral load itself (Hendley, 1998). In this context, the GM plays a critical immunomodulatory role. Depletion of beneficial bacterial genera, such as *Roseburia*, *Klebsiella*, *Coprococcus*, *Dialister (*Xu et al., 2022), *Faecalibacterium*, and *Bifidobacterium* (Hazan et al., 2022), has been associated with severe cases of COVID-19 compared to mild or moderate disease. Notably, species such as *Faecalibacterium prausnitzii*, *Eubacterium rectale*, and *Bifidobacterium adolescentis* are inversely correlated with circulating levels of proinflammatory cytokines and may exert protective effects (Yeoh et al., 2021). In contrast, increased abundances of *Prevotella*, *Streptococcus* (Zhou et al., 2023), and *Bacteroides* (Hazan et al., 2022) have been associated with more severe disease manifestations. Furthermore, GM dysbiosis may influence host lipid metabolism, which viruses exploit for replication. Elevated levels of *Blautia*, *Dorea*, *Parabacteroides*, and *Streptococcus* have been associated with the upregulation of lipid pathways and could serve as biomarkers for disease severity (Wang et al., 2023). Diet also plays a crucial role in shaping the GM and host susceptibility. Western dietary patterns, which are rich in saturated fats, sugars, and processed foods, are known to promote oxidative stress and chronic inflammation, thereby increasing proinflammatory response. Mediterranean-style diets, which are rich in fiber and fermented foods, favor the growth of SCFA-producing, anti-inflammatory bacteria that support mucosal and systemic immune regulation (Tieu et al., 2023). Additionally, microbial metabolism of dietary choline into trimethylamine N-oxide (TMAO) has been linked to an increased risk of cardiometabolic disease and potentially, to an increased susceptibility to SARS-CoV-2 infection (Tieu et al., 2023). Interestingly, patients with long-term symptoms of COVID-19, i.e., long COVID, exhibit persistent gut dysbiosis that may contribute to prolonged symptoms. These alterations include sustained depletion of SCFA-producing bacteria such as *Faecalibacterium prausnitzii* and *Bifidobacterium* spp., which compromise epithelial barrier integrity and immune regulation. This may lead to microbial translocation, chronic systemic inflammation, and immune activation, factors that are thought to underlie lingering respiratory and neurocognitive symptoms via the gut–lung and gut–brain axes (Liu et al., 2022; Gareau and Barrett, 2023). In influenza-related pneumonia, the presence of specific genera such as *Anaerotruncus*, *Barnesiella*, *Oscillibacter*, and *Cyanobacteria* has been linked to a more severe disease, suggesting a further association between GM composition and clinical outcomes (Xu et al., 2023). Taken together, these findings underscore the role of the GM in shaping the trajectory of disease, from the initial viral entry to the immune response and systemic complications.

### Asymptomatic/subclinical illness

The clinical presentation of SARS-CoV-2 infection range from asymptomatic infection to severe, life-threatening illness. Notably, up to one-third of individuals infected with SARS-CoV-2 remain asymptomatic. However, emerging evidence suggests that subclinical inflammation may still occur in these cases. For example, inflammatory changes have been detected in asymptomatic patients’ lung CT scan, indicating that the absence of symptoms does not necessarily equate to the absence of physiological effects. The host immune response appears to be a major determinant of disease severity and outcomes in COVID-19 patients. Increasing evidence indicates that the GM may modulate this immune response as certain microbial profiles may offer protection against severe inflammation, while dysbiosis can lead to uncontrolled immune activation (Zuo et al., 2020; Hussain et al., 2021). Children, who generally experience milder COVID-19 symptoms, may benefit from a more favorable GM composition. Several studies have shown that children have higher levels of butyrate-producing bacteria such as *Faecalibacterium* and *Ruminococcus*, which are associated with anti-inflammatory effects and immune modulation (Romani et al., 2022). In addition to these microbial profiles, enhanced bile acid metabolism in children may also play a protective role. Secondary bile acids have been shown to influence the differentiation of Th17 and Treg cells, contributing to immune balance (Marzano et al., 2023). Another distinguishing factor in pediatric patients is the tryptophan metabolic pathway, which appears to be upregulated in SARS-CoV-2-positive children. Tryptophan metabolites help maintain intestinal and systemic homeostasis by modulating immune activation and exerting anti-inflammatory and antioxidant effects. Interestingly, both bile acid and tryptophan metabolism are predominantly associated with the phylum *Bacillota*, further supporting the role of GM composition in shaping disease expression (Marzano et al., 2023). Although these observations are promising, further research is needed to fully elucidate the protective mechanisms involved and to clarify how gut microbial signatures influence disease progression in asymptomatic or mildly symptomatic individuals.

### Gastrointestinal manifestations

GI symptoms are frequently reported during viral respiratory infections, including those caused by SARS-CoV-2, influenza, and RSV. For SARS-CoV-2 infection, the prevalence of diarrhea has been reported to range from 4 to 25% (Cheung et al., 2020; Guan et al., 2020), while it is around 15% in patients with influenza or RSV (Newman et al., 2023). Additionally, nausea and vomiting occur in 4–20% of SARS-CoV-2 patients (Chen et al., 2022), around 35% of influenza patients, and 25% of RSV patients (Newman et al., 2023). In addition to these clinical symptoms, several studies have documented prolonged fecal shedding of SARS-CoV-2 RNA, which persists even after the virus is no longer detectable in nasopharyngeal swabs. This has been observed in both symptomatic and asymptomatic patients and appears to be unrelated to the presence or severity of GI symptoms (Zheng et al., 2020). Meta-analyses suggest that 3–31% of influenza-positive adults and 32–46% of infected children experience GI manifestations (Minodier et al., 2015). These symptoms may result from direct intestinal epithelial invasion, particularly in the case of SARS-CoV-2, which binds to ACE2 receptors expressed in enterocytes, or may arise indirectly due to systemic immune activation (Newman et al., 2023). Although the direct causal links between gut dysbiosis and GI symptoms have yet to be definitively established, alterations in the GM, especially the depletion of SCFA-producing bacteria such as *Faecalibacterium* and *Roseburia*, may contribute to GI symptoms by disrupting intestinal barrier integrity. This disruption increases gut permeability, commonly referred to as “leaky gut,” which facilitates microbial translocation and may initiate or amplify proinflammatory cascades. Consistently, elevated fecal calprotectin levels, a well-established marker of intestinal inflammation, have been observed in patients with diarrhea who have been tested positive for SARS-CoV-2 infection. This indicated the presence of underlying mucosal inflammation that may be driven by hypoxia-induced intestinal damage during severe illness (Hussain et al., 2021; Adriana et al., 2022). Importantly, in some COVID-19 cases, GI symptoms are the first, and occasionally the only, manifestation of infection (Cheung et al., 2020; Chen et al., 2022). The presence of these GI symptoms has been associated with more severe clinical outcomes, possibly reflecting a higher viral burden or more extensive systemic involvement (Newman et al., 2023; Chen et al., 2022). The frequent occurrence of GI symptoms during respiratory infections, combined with the detection of viral RNA in feces, highlights the interconnectedness of the gut and lungs via the gut–lung axis.

### Coinfections

GM dysbiosis plays a key role in immune dysregulation and disruption of intestinal barrier, thereby predisposing individuals to secondary bacterial and fungal infections during respiratory viral illnesses. In the context of SARS-CoV-2 infection, such coinfections have been associated with worse clinical outcomes (Abd El-Halim et al., 2023; Patton et al., 2024). These secondary infections can exacerbate the host’s immune response, increasing the risk of cytokine storm and extensive tissue damage (Abd El-Halim et al., 2023; Patton et al., 2024). In adult COVID-19 patients, common coinfecting pathogens include *Staphylococcus aureus*, *Streptococcus pneumoniae* (Patton et al., 2024), and *Klebsiella* species (Abd El-Halim et al., 2023). In children, *Mycoplasma pneumoniae* was frequently isolated (Patton et al., 2024). Notably, during the early months of the COVID-19 pandemic, there was a 1.84–3.14-fold increase in bloodstream infections caused by *Enterococcus* species, bacteria that are typically overrepresented in the dysbiotic GM of COVID-19 patients (Gaibani et al., 2021). These organisms are believed to translocate from the gut into the bloodstream when the intestinal barrier integrity is compromised. Similar trends have been observed in cases of influenza infection. Secondary bacterial infections are responsible for up to 40% of influenza-related deaths, and are strongly associated with higher rates of intensive care unit (ICU) admission and mortality (Qiao et al., 2023). In children with severe RSV bronchiolitis admitted to ICU, pathogenic bacteria are isolated in around 40% of cases (Thorburn et al., 2006). The mechanisms underlying this increased susceptibility include impaired mucosal defenses and reduced bactericidal activity of alveolar macrophages, which may result from decreased SCFAs production due to GM dysbiosis during viral infection (Marrella et al., 2024). This immunometabolic imbalance compromises innate immune clearance, creating a permissive environment for opportunistic pathogens. Overall, these findings emphasize the importance of maintaining gut microbial homeostasis during viral respiratory infections to prevent secondary infections and reduce disease severity.

### Dysbiosis-related extra-pulmonary comorbidities

Although respiratory viruses often present with similar pulmonary symptoms, vulnerable populations, including children, the elderly, and immunocompromised individuals, are at increased risk of severe complications, including pneumonia, acute respiratory distress syndrome, sepsis, and multi-organ failure. Importantly, patients with non-communicable diseases (NCDs) such as hypertension, cardiovascular disease, chronic kidney disease, type 2 diabetes, and obesity also experience more severe outcomes following respiratory viral infections. For example, diabetes is associated with a 7.3% higher mortality rate among COVID-19 patients (Al-Qudimat et al., 2022), and significantly increases the risk of hospitalization during influenza (threefold) and RSV (2.4–11.4-fold) infections (Branche et al., 2022; Palache et al., 2014). These chronic conditions are characterized by low-grade systemic inflammation and are often accompanied by GM dysbiosis, which further disrupts metabolic and immune homeostasis (Montanari et al., 2021). This interplay may amplify inflammatory responses and worsen disease progression during viral infections. Beyond metabolic disorders, there is growing evidence linking respiratory viral infections, particularly COVID-19 and influenza, to neuropsychiatric complications (Gallo et al., 2022; Tzang et al., 2014). Guillain–Barré syndrome is an acute and severe autoimmune disorder that affects the peripheral nervous system. It is classically associated with post-infectious immune responses, which are often triggered by GI pathogens such as *Campylobacter jejuni*, but has been linked to *Haemophilus influenzae*, *Mycoplasma pneumoniae*, and influenza viruses. More recently, a link between SARS-CoV-2 and Guillain-Barré syndrome has emerged, potentially due to viral neuroinvasion via ACE2 receptors at the blood–brain barrier and immune-mediated mechanisms (Trujillo Gittermann et al., 2020; Cao et al., 2024). During the COVID-19 pandemic, the incidence of psychosis increased by 25% compared to pre-pandemic levels (Gallo et al., 2022). Similarly, influenza infection has been linked to a sevenfold increase in the likelihood of developing schizophrenia in people with no family history of mental health issues (Tzang et al., 2014). These outcomes may be driven by neuroinflammation, with the gut–brain axis serving as a key modulator. Around 90% of afferent signals from the intestines travel to the brain, enabling the GM to influence neurodevelopment, behavior, and cognition (Gallo et al., 2022; Góralczyk-Bińkowska et al., 2022). SCFAs, particularly butyrate, play a critical role in maintaining the integrity of the blood–brain barrier and regulating neurotransmitter synthesis. Dysbiosis-associated reductions in SCFAs production may compromise these protective mechanisms, increase neuroinflammation, and exacerbate psychiatric or cognitive symptoms (Góralczyk-Bińkowska et al., 2022).

Together, these findings emphasize the widespread impact of gut microbial dysbiosis during respiratory infections, affecting not only the lungs, but also metabolic, neurological, and psychiatric health. This highlights the importance of preserving GM integrity in vulnerable populations to prevent extrapulmonary complications.

## Gut microbiota and vaccines: a bidirectional interaction

Vaccination is one of the most effective public health tools for preventing infectious diseases, saving an estimated 2–3 million lives each year (Lynn et al., 2022; Gonçalves et al., 2022). However, the efficacy of vaccine can vary significantly from person to person, with factors such as age, geography, and GM composition influencing the immune response. The relationship between the GM and vaccine response is bidirectional: vaccines can alter GM composition, while the GM, in turn, affects vaccine efficacy and side effects. For example, individuals in low- to middle-income countries, as well as infants and the elderly, often exhibit weaker responses to vaccines than other groups (Lynn et al., 2022). Several studies have demonstrated associations between GM composition and vaccine immunogenicity in the context of SARS-CoV-2. In immunocompetent individuals, high abundances of *Bifidobacterium adolescentis* and *Roseburia faecis* have been linked to stronger neutralizing antibody responses. Similarly, in immunocompromised individuals, *Bilophila*, *Alistipes*, and *Butyricicoccus* were positively correlated with antibody titers. In contrast, elevated levels of *Streptococcus* and *Parabacteroides* were associated with weaker serological responses (Ng et al., 2022; Alexander et al., 2023). Furthermore, individuals with higher levels of SCFAs, which play a key role in B cell metabolism and antibody production, tend to exhibit enhanced immune responses to vaccination. SCFAs fuel oxidative phosphorylation, glycolysis, and fatty acid synthesis in immune cells, thereby supporting efficient humoral immunity (Lynn et al., 2022; Alexander et al., 2023). GM composition also appears to influence the adverse effects experienced following vaccination. Individuals who reported fewer side effects after vaccination showed an increase in *Prevotella copri* and two *Megamonas* species, which are believed to have anti-inflammatory properties (Ng et al., 2022). Conversely, vaccines may also induce transient disruptions in gut microbial homeostasis. For example, SARS-CoV-2 vaccination has been linked to a temporary decrease in alpha diversity, and shifts in microbial composition, such as a decrease in *Bacteroidota* and *Pseudomonadota*, and an increase in *Bacillota* (Jiao et al., 2022; Tang et al., 2022). These changes include an increase in beneficial taxa such as *Faecalibacterium*, *Bifidobacterium*, *Lachnospira*, *Roseburia*, *Anaerostipes hadrus*, *Ruminococcus torques*, and *Oscillibacter*, alongside a decrease in *Bacteroides* and *Blautia* (Jiao et al., 2022; Tang et al., 2022). These findings highlight the potential for targeted modulation of the GM to enhance vaccine efficacy. Strategies to optimize vaccine-induced protection, particularly in vulnerable populations such as the elderly, infants, and immunocompromised patients, could include prebiotics, probiotics, or dietary interventions aimed at supporting beneficial microbial communities.

## Modulation of gut microbiota in the prevention and treatment of infections

Due to strong association between intestinal dysbiosis and disease progression in respiratory viral infections, various strategies have been suggested to restore microbial balance and improve clinical outcomes. Among these, the use of prebiotics and probiotics, for both prophylactic and therapeutic purposes, has gained considerable attention. Probiotics exert their effects through both direct and indirect mechanisms. Directly, they can trap viruses, inhibit replication, and produce antiviral compounds such as bacteriocins. Indirectly, they modulate the host immune response by enhancing the production of interleukins, IgA, and by improving the function of CD4 + and CD8 + T cells and natural killer cells. Certain strains have also been shown to promote the growth of beneficial taxa such as *Faecalibacterium prausnitzii*, *Akkermansia*, and *Lactobacillus*. Importantly, these effects are strain-specific and are not limited to live bacteria. Even non-viable probiotics or bacterial components such as polysaccharides can exert immunomodulatory properties (Mirzaei et al., 2021; Shinde et al., 2020). Preclinical and clinical studies suggest that probiotics may help prevent upper respiratory tract infections, reduce symptom duration, and modulate inflammatory responses (Mirzaei et al., 2021), though results remain inconsistent across studies (Lehtoranta et al., 2014). A recent meta-analysis of 18 trials involving patients with SARS-CoV-2 infection showed that probiotics, primarily *Bifidobacterium* and *Lactobacillus* strains, were associated with reduced mortality, shorter hospital stays, faster recovery, and lower rates of clinical deterioration, whether administered alone or in combination with standard therapy (Sohail et al., 2023). However, the heterogeneity of probiotic formulations, dosing regimens, and study populations, along with variations in study quality, limits the generalizability of these findings. Large-scale randomized controlled trials are needed to establish consistent efficacy and optimal protocols. Moreover, the current evidence base does not support the use of probiotics in the prevention of SARS-CoV-2 infection, highlighting the need to define their optimal indications (Sohail et al., 2023). Certain probiotic-derived compounds, such as those from *Lactobacillus plantarum*, have demonstrated *in silico* the ability to prevent SARS-CoV-2 from entering into host cells (Batista et al., 2023; Anwar et al., 2020). Probiotics have also shown promise as vaccine adjuvants or delivery vectors, enhancing seroconversion and strengthening immune responses to respiratory virus vaccines (Gonçalves et al., 2022). However, to realize their full therapeutic potential, it is essential to standardize the choice of bacterial strains, dosage, and administration protocols across studies. In summary, prebiotics and probiotics are promising adjuvant strategies for preventing and treating respiratory viral illnesses. While still in the experimental phase, their use may complement traditional therapies and support host immune defenses, especially in populations with known dysbiosis or impaired vaccine responses.

## Concluding remarks

Respiratory viral infections such those caused by SARS-CoV-2, influenza, and RSV have systemic effects that extend well beyond the lungs. One of the most significant effects is the disruption of GM composition, which may persist even after recovery. The gut-lung axis plays a central role in this bidirectional relationship: pulmonary infections can disturb the gut microbial balance, while gut dysbiosis can, in turn, exacerbate the disease (Enaud et al., 2020; Zhou et al., 2021). These alterations are primarily characterized by a decrease in beneficial, including SCFA-producing bacteria, and an increase in opportunistic pathogens, which contribute to intestinal permeability and systemic inflammation (Zuo et al., 2020; Gaibani et al., 2021; Nagata et al., 2023; Qin et al., 2015; Harding et al., 2020). Although there is no direct evidence linking gut dysbiosis to GI symptoms during respiratory infections, GM composition appears to be a key factor in modulating disease progression and severity. This can potentially increase individuals’ susceptibility to coinfections and the onset of NCDs, and may contribute to the development of neuropsychiatric symptoms. GM imbalances following these infections can trigger a proinflammatory state, leading to increased gut permeability and subsequent GI manifestations reported in patients (Hussain et al., 2021). The consequences of intestinal dysbiosis extend beyond GI symptoms, to influence immune responses, viral entry, and extrapulmonary complications, thereby worsening disease severity. Given the significant roles of GM, therapeutic strategies aiming at restoring microbial balance could provide a new way of preventing and managing these infections. Although probiotics and prebiotics have shown promise in enhancing antiviral immunity, standardized and large-scale confirmatory studies are needed to ensure consistent outcomes (Mirzaei et al., 2021; Shinde et al., 2020; Sohail et al., 2023). While these strategies are still experimental, they demonstrate the potential of microbiome-targeted interventions in respiratory viral infections. Furthermore, microbiota composition has been linked to immune responses to vaccines, with for instance *Bifidobacterium* and *Roseburia* enhancing antibody production (Ng et al., 2022; Alexander et al., 2023). Therefore, microbiota-targeted interventions could optimize vaccine responses. Overall, the GM emerges as a key player in the pathogenesis of viral infections such as COVID-19, influenza, and RSV, influencing not only disease severity, but also recovery and vaccine responses. Future research should focus on longitudinal assessments of microbiota alterations and host-microbiota interactions, as well as the development of targeted microbiome-based therapies to mitigate the impact of viral respiratory infections. Gaining deeper insight into these complex relationships may open new approaches in personalized medicine, optimizing immune resilience, and improving clinical outcomes.

## Glossary

ACE2: Angiotensin Converting Enzyme 2
BALT: Bronchial-Associated Lymphoid Tissue
COVID-19: Coronavirus Disease 2019
GALT: Gut-Associated Lymphoid Tissue
GI: Gastrointestinal
GM: Gut Microbiota
ICU: Intensive Care Unit
Ig: Immunoglobulin
LPS: Lipopolysaccharide
MALT: Mucosa-Associated Lymphoid Tissue
MAMPs: Microbial-Associated Molecular Patterns
NCD: Non-Communicable Disease
RSV: Respiratory Syncytial Virus
SARS-CoV: Severe Acute Respiratory Coronavirus 1
SARS-CoV-2: Severe Acute Respiratory Coronavirus 2
SCFA: Short Chain Fatty Acid
Th: T helper cells
TLR: Toll-Like Receptor
TMAO: Trimethylamine N-oxide
Treg: Regulatory T cells
